# Autoantibodies and autoantigens in autoimmune hepatitis: important tools in clinical practice and to study pathogenesis of the disease

**DOI:** 10.1186/1740-2557-1-2

**Published:** 2004-10-15

**Authors:** Kalliopi Zachou, Eirini Rigopoulou, George N Dalekos

**Affiliations:** 1 Research Laboratory of Internal Medicine, Department of Medicine, Larissa Medical School, University of Thessaly, Larissa 41222, Greece; 2 Academic Liver Unit, Department of Medicine, Larissa Medical School, University of Thessaly, Larissa 41222, Greece

**Keywords:** Antibodies against Liver Cytosol 1 Antigen (anti-LC1), Antibodies against Soluble Liver Antigens or Liver Pancreas (anti-SLA/LP), Antinuclear Antibodies (ANA), Antineutrophil Cytoplasmic Autoantibodies (ANCA), Autoimmune Hepatitis, Cytochrome P450 2D6, Cytochrome P450 2A6, Cytochrome P450 1A2, Hepatitis C, Hepatitis D, Liver-Kidney Microsomal Autoantibodies (anti-LKM), Liver Microsomal Autoantibodies (anti-LM), Smooth Muscle Autoantibodies (SMA)

## Abstract

Autoimmune hepatitis (AIH) is a chronic necroinflammatory disease of the liver characterized by hypergammaglobulinemia, characteristic autoantibodies, association with HLA DR3 or DR4 and a favorable response to immunosuppressive treatment. The etiology is unknown. The detection of non-organ and liver-related autoantibodies remains the hallmark for the diagnosis of the disease in the absence of viral, metabolic, genetic, and toxic etiology of chronic hepatitis or hepatic injury. The current classification of AIH and the several autoantibodies/target-autoantigens found in this disease are reported. Current aspects on the significance of these markers in the differential diagnosis and the study of pathogenesis of AIH are also stated. AIH is subdivided into two major types; AIH type 1 (AIH-1) and type 2 (AIH-2). AIH-1 is characterized by the detection of smooth muscle autoantibodies (SMA) and/or antinuclear antibodies (ANA). Determination of antineutrophil cytoplasmic autoantibodies (ANCA), antibodies against the asialoglycoprotein receptor (anti-ASGP-R) and antibodies against to soluble liver antigens or liver-pancreas (anti-SLA/LP) may be useful for the identification of patients who are seronegative for ANA/SMA. AIH-2 is characterized by the presence of specific autoantibodies against liver and kidney microsomal antigens (anti-LKM type 1 or infrequently anti-LKM type 3) and/or autoantibodies against liver cytosol 1 antigen (anti-LC1). Anti-LKM-1 and anti-LKM-3 autoantibodies are also detected in some patients with chronic hepatitis C (HCV) and chronic hepatitis D (HDV). Cytochrome P450 2D6 (CYP2D6) has been documented as the major target-autoantigen of anti-LKM-1 autoantibodies in both AIH-2 and HCV infection. Recent convincing data demonstrated the expression of CYP2D6 on the surface of hepatocytes suggesting a pathogenetic role of anti-LKM-1 autoantibodies for the liver damage. Family 1 of UDP-glycuronosyltransferases has been identified as the target-autoantigen of anti-LKM-3. For these reasons the distinction between AIH and chronic viral hepatitis (especially of HCV) is of particular importance. Recently, the molecular target of anti-SLA/LP and anti-LC1 autoantibodies were identified as a 50 kDa UGA-suppressor tRNA-associated protein and a liver specific enzyme, the formiminotransferase cyclodeaminase, respectively. Anti-ASGP-R and anti-LC1 autoantibodies appear to correlate closely with disease severity and response to treatment suggesting a pathogenetic role of these autoantibodies for the hepatocellular injury. In general however, autoantibodies should not be used to monitor treatment, predict AIH activity or outcome. Finally, the current aspects on a specific form of AIH that may develop in some patients with a rare genetic syndrome, the autoimmune polyendocrinopathy-candidiasis-ectodermal dystrophy syndrome (APECED) are also given. Autoantibodies against liver microsomes (anti-LM) are the specific autoantibodies detected in AIH as a disease component of APECED but also in cases of dihydralazine-induced hepatitis. Cytochrome P450 1A2 has been identified as the target-autoantigen of anti-LM autoantibodies in both APECED-related AIH and dihydralazine-induced hepatitis. The latter may indicate that similar autoimmune pathogenetic mechanisms can lead to liver injury in susceptible individuals irrespective of the primary defect. Characterization of the autoantigen-autoantibody repertoire continues to be an attractive and important tool to get access to the correct diagnosis and to gain insight into the as yet unresolved mystery of how hepatic tolerance is given up and AIH ensues.

## 1. Introduction

Autoimmune hepatitis (AIH) is a rare chronic liver disease of unknown etiology. The estimated prevalence of AIH in Northern European countries is approximately 160–170 patients/10^6 ^inhabitants [1,2]. The disease predominates among women and is characterized by hypergammaglobulinemia even in the absence of cirrhosis, characteristic autoantibodies, association with human leukocyte antigens (HLA) DR3 or DR4 and a favorable response to immunosuppressive treatment [3-5]. The onset of AIH disease is usually insidious, with unspecific symptoms, such as, fatigue, malaise, arthralgias, and fluctuating jaundice, right upper quadrant pain or lethargy [5-8]. However, a substantial proportion of patients may have no obvious signs or symptoms of liver disease, while occasionally the presentation may be severe and almost identical to an acute or fulminant episode of viral hepatitis [5-8]. Although AIH brings in mind the archetypal patient being a young female with endocrine abnormalities, there is nowadays increasing evidence that the disease can also affect males and can present at almost any age (the large majority of patients being between 50 and 70 years of age) [6-12].

Liver histology is not pathognomonic for AIH and there is no single serologic test of sufficient specificity for the diagnosis of AIH as for the diagnosis of viral hepatitis A to E. Although the presence of autoantibodies is one of the distinguishing features of AIH, there is no single autoantibody with the diagnostic significance and specificity that antimitochondrial autoantibodies (AMA) demonstrate for the diagnosis of primary biliary cirrhosis (PBC). For this reason, autoantibodies can not be employed as a single marker for the diagnosis of AIH. It is rather a diagnosis reached by the exclusion of other factors leading to chronic hepatitis that include viral, toxic, genetic and metabolic causes [6]. Under this context, it is clear that sometimes AIH may be difficult to diagnose [7,8]. In 1992, the International Autoimmune Hepatitis Group reported a descriptive set of criteria that could be applied in the routine clinical practice for the diagnosis and classification of patients as having either 'definite' or 'probable' AIH [13]. In addition, a diagnostic scoring system was devised to provide an objective method for selection of relatively homogeneous groups of patients for research purposes [13]. The same group has remarkably simplified the descriptive set of criteria and the diagnostic scoring system in late 1998 (Tables 1 and 2) [6]. The diagnostic score demonstrates that the presence of defined autoantibodies is an integral part of the diagnosis of AIH but not its single diagnostic tool [6,14].

**Table 1:** Revised Scoring System for the Diagnosis of Autoimmune Hepatitis^6^.

**Parameter/Features**	**Score**
**Gender**	
- Female/Male	+2/0
**Degree of elevation above upper normal limit of ALP vs. aminotransferases**	
- <1.5	+2
- 1.5 – 3.0	0
- >3.0	-2
**Total serum globulins, γ-globulins, or IgG above normal**	
- >2.0	+3
- 1.5 – 2.0	+2
- 1.0 – 1.5	+1
- <1.0	0
**ANA, SMA or LKM-1 (titers by immunofluorescence on rodent tissues or HEp2-cells)**	
- >1 : 80	+3
- 1 : 80	+2
- 1 : 40	+1
- <1 : 40	0
- AMA positive	-4
**Hepatitis viral markers (IgM anti-HAV, HBsAg, IgM anti-HBc, anti-HCV and HCV-RNA)**	
- Positive/Negative	-3/+3
**Recent or current use of known or suspected hepatotoxic drugs**	
- Yes/No	-4/+1
**Average alcohol intake**	
- <25 g/day	+2
- >60 g/day	-2
**Other autoimmune disease(s) in patient or first degree relatives**	
- Yes/No	+2/0
**Optional additional parameters (should be allocated only if ANA, SMA or LKM-1 are negative)**	
- HLA DR3, DR4, or other HLA with published association with AIH)	+1
- Seropositivity for any of ANCA, anti-LC1, anti-SLA/LP, anti-ASGPR and anti-sulfatide	+2
**Liver histology**	
- Interface hepatitis	+3
- Predominant lymphoplasmacytic infiltrate	+1
- Rosetting of liver cells	+1
- None of the above	-5
- Biliary changes	-3
- Other changes	-3
**Response to therapy (as defined in Table 2)**	
- Complete/Relapse	+2/+3

Definite AIH if greater than 15 before treatment or greater than 17 post-treatment; probable AIH if varies between 10–15 before treatment or 12–17 post-treatment. ALP: alkaline phospatase, IgM anti-HAV: hepatitis A virus IgM antibody, anti-HCV: hepatitis C virus antibody, HBsAg: surface antigen of hepatitis B virus, IgM anti-HBc: IgM antibody against the nuclear antigen of hepatitis B virus. Other abbreviations are the same as shown in the text.

**Table 2:** Definitions of Response to Therapy.

**Response**	**Definition**		
Complete	Either or both of the following: marked improvement of symptoms and return of serum AST or ALT, bilirubin and immunoglobulin values completely to normal within 1 year and sustained for at least a further 6 months on maintenance therapy, or liver biopsy specimen at some time during this period showing at most minimal activity.	or	Either or both of the following: marked improvement of symptoms together with at least 50% improvement of all liver tests during the first month of treatment, with AST or ALT levels continuing to fall to less than twice the upper normal limit within 6 months during any reductions toward maintenance therapy, or a liver biopsy within 1 year showing only minimal activity.
Relapse	Either or both of the following: an increased in serum AST or ALT levels of greater than twice the upper normal limit or a liver biopsy showing active disease, with or without reappearance of symptoms, after a "complete" response as defined above.	or	Reappearance of symptoms of sufficient severity to require increased (or reintroduction of) immunosuppression, accompanied by any increase in serum AST or ALT levels, after a "complete" response as defined above.

AST: aspartate aminotransferase, ALT: alanine aminotransferase.

In recent years however, significant progress has been made in the characterization of liver-related target-autoantigens. This has led to the notion that some of the major target-autoantigens in AIH are active enzymes of the human hepatic and non-hepatic microsomal xenobiotic metabolism [14-16]. The latter serve as a means to investigate this still enigmatic liver disease. This article will focus on the data that have evolved in the course of the characterization of autoantibody-autoantigen "system" in AIH by giving the current aspects on the role and significance of this "system" in the differential diagnosis and study of pathogenesis of AIH.

## 2. Classification of AIH

According to the pattern of autoantibodies detected in AIH patients, a subclassification of the disease into three types was proposed in 1994 [17]. AIH type 1 (AIH-1) is characterized by the presence of antinuclear antibodies (ANA) and/or smooth muscle autoantibodies (SMA) which may associate with perinuclear anti-neutrophil cytoplasmic antibodies (p-ANCA) [3,5,6,14,15]. AIH type 2 (AIH-2) is characterized by the detection of specific autoantibodies against liver and kidney microsomal antigens (anti-LKM type 1 or infrequently type 3) [14-16,18] and/or antibodies against liver cytosol type 1 antigen (anti-LC1) [14,15,19]. AIH type 3 (AIH-3) is characterized by autoantibodies against soluble liver antigens (anti-SLA) [20] or to liver-pancreas antigen (anti-LP) [21,22].

The serological diversity of autoantibodies found in AIH supports the aforementioned subclassification and provides a framework for the scientific analysis of this heterogeneous disease group [5,15]. It also demonstrates that AIH may not be a single disease with a single underlying mechanism but most likely is a group of diseases with a similar clinical presentation [14,15]. This is further substantiated by the finding of an unusual form of AIH in 10–18% of patients with a rare autosomal recessive disorder, the autoimmune polyendocrinopathy-candidiasis-ectodermal dystrophy syndrome (APECED) [23-25]. This syndrome is characterized by chronic mucocuteneous candidiasis, ectodermal dystrophy and autoimmune tissue destruction particularly of the endocrine glands (hypoparathyroidism, adrenocortical failure and gonadal failure in females) [26-29].

However, due to recent clinical, serologic and genetic findings, it has been suggested that anti-SLA seropositive patients do not define a subgroup of AIH, but rather belong to the AIH-1 group [30-32]. For this reason, subdivision into AIH-1 (ANA, SMA, p-ANCA and/or anti-SLA/LP positive) and AIH-2 (anti-LKM-1, anti-LKM-3 and/or anti-LC1 positive) is in common usage (Table 3). Apart from serological differences, AIH-2 seems to be clinically and genetically distinguishable from AIH-1 [5,8,14]. Indeed, patients with AIH-2 are younger at presentation, usually have higher levels of bilirubin and transaminases, and are characterized by more severe disease than patients with AIH-1 [5,6,14,18,33]. In addition, contrary to what has been recorded in patients with AIH-1, no sustained remission has been observed after the discontinuation of immunosuppressive therapy in patients with AIH-2 [4,33]. Taking into consideration the genetic markers, it has been found that the association between HLA DR3 and AIH-2 is rather weaker than that reported in AIH-1, while an association between HLA DQ2 and AIH-2 has been reported [14,18,33,34]. However, AIH-2 patients represent only a small proportion of the total cases of AIH [3-6]. In addition, the long-term outcome of the affected patients appears to be similar both in AIH-1 and AIH-2 [6,33]. Therefore, the classification of AIH in these two major subgroups is still uncertain and controversial [6,8,13].

**Table 3:** Classification of Autoimmune Hepatitis (AIH) According to Autoantibodies Detection

*Type of AIH*	*Characteristic autoantibodies*
AIH-1	ANA, SMA, p-ANCA anti-ASGP-R, anti-SLA/LP
AIH-2	anti-LKM-1, anti-LKM-3, anti-LC1, anti-ASGP-R

Abbreviations are the same as shown in the text.

## 3. Detectable Autoantibodies in AIH-1

### 3.1. Anti-nuclear antibodies (ANA) and smooth muscle autontibodies (SMA)

ANA and/or SMA are almost exclusively requisites for the diagnosis of AIH-1 [3,5,6,14,15,30]. In typical cases of AIH-1, these autoantibodies are detected in significant titers (≥1;80 in adults and ≥1:40 in children) in almost half of Caucasians patients with AIH-1, while ANA alone are detected in 15% and SMA alone in 35% [5,30,35].

**Figure 1:**
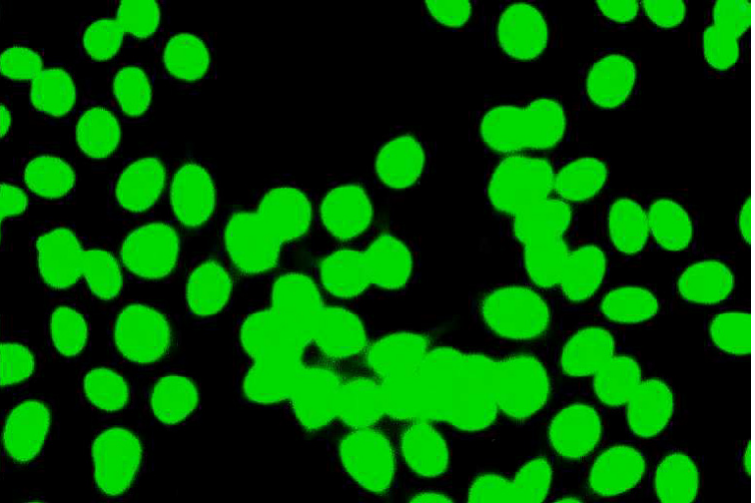
High titer antinuclear antibodies (ANA) of the homogeneous pattern by indirect immunofluoerescence on immobilized HEp-2 cells in a female patient with autoimmune hepatitis type 1 (AIH-1). Homogeneous ANA are frequently found in AIH-1 (original magnification 40×).

**Figure 2:**
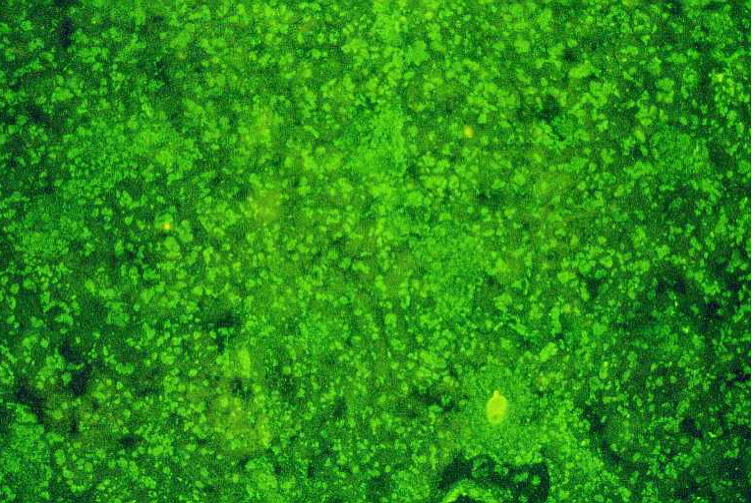
Typical staining of antinuclear antibodies in the serum of a patient with autoimmune hepatitis type 1 visualized by indirect immunofluoerescence on cryostat sections of rat liver (original magnification 40×).

The most frequent and conventional method for ANA detection is the indirect immunofluorescence (IIF) assay on cryostat sections of rodent tissues and HEp-2 cells slides (Figures 1 and 2) [14,15,35,36]. Different patterns of fluorescence are found by this assay due to the large variability of target-autoantigens in the nuclei of HEp-2 cells that have been recognized [35-40]. Actually, ANA are found to be directed against single or double stranded DNA, tRNA, SSA-Ro, snRNPs, laminins A and C, cyclin A or histones [35-40]. Most commonly, a homogenous (34–58%) or speckled (21–34%) immunofluoerescence pattern is demonstrable [14,15,35-37]. So far however, neither a liver-specific nuclear antigen nor a liver-disease-specific ANA has been identified. For this reason, subtyping of ANA in cases of AIH-1 seems to have limited clinical implication and diagnostic relevance in routine clinical practice [6,14,15,35-40].

**Figure 3:**
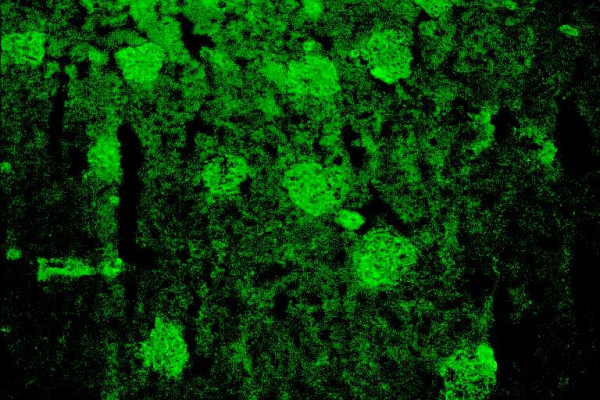
Smooth muscle antibodies by indirect immunofluoerescence on rat kidney (from a female patient with autoimmune hepatitis type 1). The immunofluorescence involves smooth muscle fibers within blood vessels (original magnification 40×).

**Figure 4:**
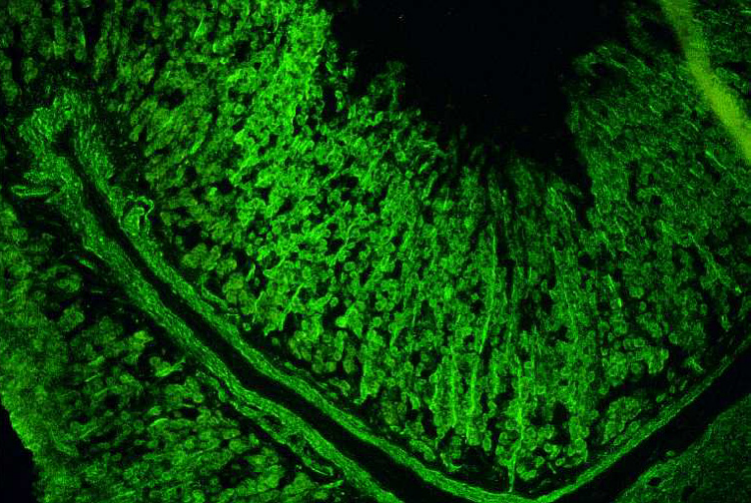
Smooth muscle autoantibodies by indirect immunofluoerescence on rat stomach (serum from a female patient with autoimmune hepatitis type 1). The immunofluorescence involves smooth muscle fibers within muscularis mucosa (original magnification 40×).

SMA are detected by IIF on rodent liver and kidney, due to staining of vessel walls (Fig. 3), and stomach due to staining of the muscle layer (Fig. 4). SMA are directed against structures of the cytoskeleton such as actin, troponin, vimentin and tropomyosin. In AIH-1, SMA are predominantly directed against F-actin [41]. The latter seems to be diagnostically more relevant in pediatric patients where SMA may be the only marker of AIH-1 even in titers as low as of 1:40 [14,15]. Czaja et al [41] have suggested that antibodies to actin are associated with a younger age of disease onset, the presence of HLA-A1-B8-DR3 haplotype and a greater frequency of treatment failure, death from liver disease and earlier requirement for transplantation than actin-antibody negative AIH-1 patients.

ANA and/or SMA – usually in low titers – may occur in patients with chronic viral hepatitis B or C, but in most of these cases SMA lack F-actin specificity [14,15,42,43]. From the clinical point of view, interferon-alpha administration is generally safe in most cases of viral hepatitis with ANA and/or SMA, although occasionally may provoke mild self-limited autoimmune disorders compared to viral hepatitis patients without ANA or SMA autoantibodies [44-46]. During immunosuppressive treatment, disappearance of ANA and/or SMA is observed in the majority of patients with AIH-1 [47]. However, autoantibody status is unable to predict immediate outcome after cessation of corticosteroid administration. Additionally, neither autoantibody titers at first diagnosis nor autoantibody behaviour in the time course of the disease are prognostic markers for AIH-1 [14,15,47]. These findings indicate that ANA and SMA are not involved in the pathogenesis of AIH-1 and furthermore, their determination is more of diagnostic than prognostic value [5,14,15,47].

### 3.2. Anti-neutrophil cytoplasmic autoantibodies (ANCA)

These autoantibodies are directed against cytoplasmic constituents of neutrophil granulocytes and monocytes. Classically, they are detected by IIF using ethanol-fixed granulocytes as substrate [48]. Using the above method, two major subtypes can be distinguished. ANCA showing a diffuse or granular cytoplasmic staining (c-ANCA) and ANCA characterized by a perinuclear-staining (p-ANCA). Both c-ANCA and p-ANCA are valuable diagnostic and prognostic markers in systemic vasculitides in particular Wegener's granulomatosis and microscopic polyangiitis, respectively [48,49]. Proteinase-3 has been identified as the major target-autoantigen of c-ANCA in cases with Wegener's granulomatosis, while myeloperoxidase is the documented autoantigen of p-ANCA in most patients with microscopic polyangiitis [48,49]. Since then, ANCA (in most cases of p-ANCA type) were detected in a high prevalence in other inflammatory disorders of unknown aetiology such as, inflammatory bowel disease (more frequently in ulcerative colitis than in Crohn's disease) [50,51] and primary sclerosing cholangitis (PSC), a liver disease that is frequently associated with ulcerative colitis [51,52].

**Figure 5:**
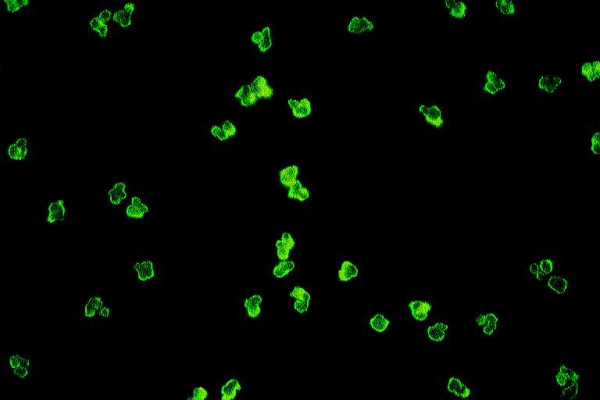
Perinuclear staining of anti-neutrophil cytoplasmic autoantibodies (p-ANCA) by indirect immunofluoerescence on ethanol fixed human granulocytes (serum from an ANA negative patient with autoimmune hepatitis type 1). Original magnification 40×).

Several recent studies however, have also documented the presence of high titers of p-ANCA in the sera of patients with AIH-1 (Fig. 5; prevalence range 40–96%) [53-58] and to a much lesser extent in PBC patients (prevalence range 0–39%) [54,58,59]. Occasionally, high titers of c-ANCA can be detected in AIH-1 (Dalekos GN, 2002, unpublished observations). In contrast, p-ANCA have not been detected in serum samples from patients with AIH-2 [57]. Low ANCA titers are detected infrequently in patients with alcoholic or chronic viral liver diseases [54,57,60]. However, a recent large study in 516 patients with hepatitis C virus (HCV) infection revealed the presence of ANCA in as high as 55.6% of patients [61]. Interestingly these investigators have shown that all HCV positive sera with ANCA had c-ANCA pattern on IIF and contained proteinase-3 specificity [61]. The clinical relevance of this finding remains to be determined. In patients with AIH-1, PBC or PSC the detection of ANCA appears to be associated with a more severe disease course or the presence of cirrhosis [54,62]. The latter suggestion however, was not confirmed by more recent studies [53,54].

To determine the antigenic specificities of ANCA, antigen-specific enzyme linked immunosorbent assays (ELISAs) and Western blotting followed by immunodetection can be performed [14,63]. Using these techniques it became obvious that in AIH-1 the target-autoantigens recognized are multiple including cathepsin G, catalase, alpha-enolase, lactoferrin, actin and high mobility group (HMG) non-histone chromosomal proteins HMG1 and HMG2 [14,54,56,58,62-65]. However, the determination of antigenic specificities of ANCA seems to have limited clinical relevance in patients with AIH-1 [14,15,54,56].

In conclusion, the detection of ANCA may be a useful additional marker in searching for AIH-1, in particular in ANA/SMA/anti-LKM-1 negative cases of AIH. With the exception of a recent paper by Wu et al [61] the detection of ANCA is rather rare in chronic viral hepatitis [14,54,57,60]. The latter may prove useful in the differential diagnosis between patients with AIH and those with viral hepatitis who tested positive for ANA or SMA. Furthermore, since ANCA appear to be relatively rare in PBC [54,59], these autoantibodies may prove useful for distinguishing between genuine cases of AIH and cases of PBC with features overlapping with those of AIH [6]. However, due to the lack of specificity for the diagnosis of AIH and to its obscure role – if any – in AIH, their routine determination is not recommended [14,15].

### 3.3. Autoantibodies against the asialoglycoprotein receptor (anti-ASGP-R)

The asialoglycoprotein receptor (ASGP-R) is a liver-specific glycoprotein of the cell membrane. Its main function is the internalization of asialoglycoproteins by binding a terminal galactose residue to coated pits. Anti-ASGP-R autoantibodies are detected in 88% of patients with AIH (both types) [66,67]. However, these autoantibodies are also found in some patients with PBC, chronic viral hepatitis B and C and alcoholic liver disease although at lower frequency and lower titers [14,15,66,67].

The ASGP-R is preferentially expressed on the surface of periportal liver cells where piecemeal necrosis is found as a marker of severe inflammatory activity in patients with AIH [68]. This finding may suggest a possible immunopathogenetic involvement of anti-ASGP-R autoantibodies in AIH [69]. The general presumption is that target of potentially tissue-damaging autoreactions in AIH must be liver-specific and available to the immune system in vivo (e.g. expression on the surface of hepatocytes). So far, ASGP-R is the only target-autoantigen that has been positively identified and fulfils these criteria [68,69]. Additional support to this emerged from the determinations of anti-ASGPR autoantibodies in consecutive AIH patients. The levels of anti-ASGP-R autoantibodies vary according to the inflammatory activity of the disease. In addition, anti-ASGP-R antibody titers decreased significantly in response to immunosuppression, while they reappear when the disease has relapsed [66,70]. These autoantibodies may be diagnostically helpful when other autoantibodies are not detected and AIH is suspected. However, due to the belief that anti-ASGPR antibody represents a general marker of liver autoimmunity and the limitations in its detection (requires chemically purified ASGP-R, which is not yet widely available), its routine use is not generally recommended.

### 3.4. Antibodies against soluble liver antigens (anti-SLA) or to liver-pancreas antigen (anti-LP)

The anti-SLA autoantibodies were described for the first time in 1987 [20]. They cannot be detected by IIF on common substrate. A competitive ELISA or a radioimmunoassay usually detects these autoantibodies [20,32,71]. SLA is found in 100000 g supernatant of liver homogenate and represent a cytosolic protein which is neither organ nor species specific [72]. However, the highest concentrations are found in liver and kidney tissues. The anti-SLA autoantibodies are detected in patients with AIH alone or in combination with SMA and/or ANA [30-32,73]. As noted above, similarities in the clinical profile between patients with AIH-1 (ANA and/or SMA positive) and AIH patients with anti-SLA alone in addition with an approximately 30% seropositivity overlap between anti-SLA and SMA and/or ANA suggest that anti-SLA is rather an additional important marker for the diagnosis of AIH-1, than a marker of a third type of AIH [6,14,30-32].

A scientific group from Tuebingen, Germany described for the first time the anti-LP autoantibodies in 1981 [21]. The LP antigen was predominantly detected in the S100 supernatant of liver and pancreas homogenates, indicating that this antigen was a soluble protein. Until recently, anti-LP and anti-SLA autoantibodies were thought to be different [20-22]. However, Wies et al report [74] provides convincing evidence and confirms previous suggestions that anti-SLA and anti-LP are one and the same autoantibody (anti-SLA/LP). In addition, the same study demonstrated that the identified target-autoantigen of anti-SLA/LP autoantibodies (a 35–50 kDa protein) was neither cytokeratins 8 or 18 [71] nor glutathione-S-transferase isoenzyme [75]. The results from two independent groups [76,77] were similar with those found by Wies et al [74]. After screening of cDNA expression libraries they identified a previously unknown amino acid sequence, which presumably encodes a UGA-suppressor tRNA-associated protein, as the targen-autoantigen of anti-SLA/LP autoantibodies [76,77]. The UGA-suppressor serine tRNA-protein complex is likely to be involved in cotranslational selenocysteine incorporation in human cells [78]. It was then obvious that the identification of SLA/LP autoantigen would allow the establishment of a reliable, universally available diagnostic test for AIH but also it would provoke the investigation in the area of autoimmune liver diseases.

Regarding disease specificity, anti-SLA/LP autoantibodies have not been detected in patients with AIH-2, PBC, PSC, chronic viral hepatitis, alcoholic liver disease and non-hepatic autoimmune diseases by standardized ELISAs using reference autoantibody or recombinant antigen [20,32,73,79]. Ballot et al [32] also showed that these autoantibodies are different from anti-LC1. For these reasons, anti-SLA/LP has been considered as a valuable and specific diagnostic marker of AIH [31,32,73,74,76,77,79]. However, a recent study from the United Kingdom [80] has shown that anti-SLA/LP autoantibodies can also be detected in AIH-2 and in children with PSC. These investigators used eukaryotically expressed tRNP ((Ser) Sec)/SLA as target in a radioligand assay (RLA) which is well known as a more sensitive test than ELISAs and immunoblot due to its ability to identify antibodies directed to conformational epitopes [81-83]. Their novel findings need confirmation from other research groups and particularly to address whether anti-SLA/LP reactivity is also present in adult PSC. Recent data confirmed the previous finding that patients with anti-SLA/LP display a more severe course of AIH [79,80,84]. The latter suggest that anti-SLA/LP may be linked to the pathogenesis of the autoimmune process although the exact function and its role in autoimmunity are so far unclear [14,15]. From the clinical point of view however, this autoantibody may be helpful in an attempt to reduce the group of cryptogenic hepatitis and/or cirrhosis.

## 4. Detectable Autoantibodies in AIH-2

### 4.1. Autoantibodies against liver-kidney microsomes (anti-LKM)

**Figure 6:**
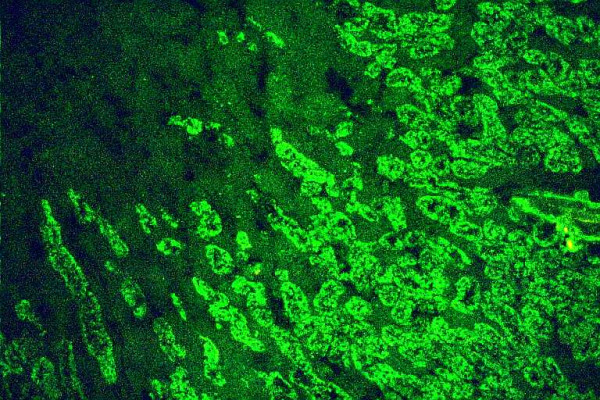
Antibodies against liver-kidney microsomes type 1 (anti-LKM-1) react to the proximal tubules of the rat kidney. The absence of reactivity against thedistal tubules of the rat kidney (see also Fig. 6B) and parietal cells of the rat stomach distinguishes anti-LKM-1 autoantibodies from antimitochondrial antibodies (original magnification 40×).

**Figure 7:**
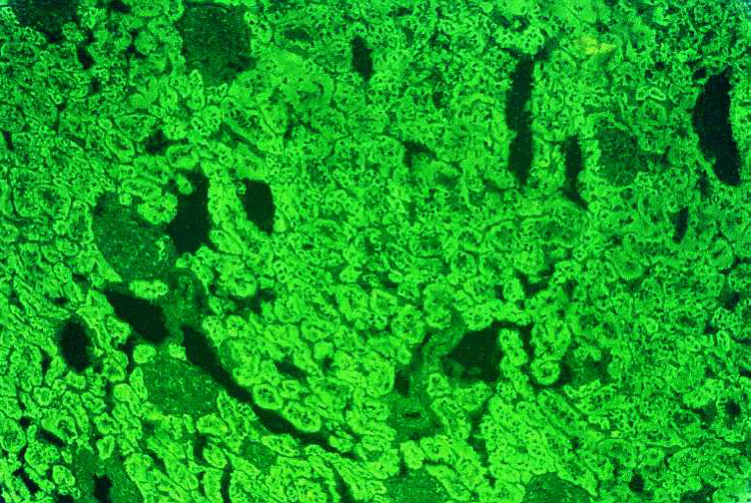
Antimitochondrial antibodies react to the proximal and distal tubules of the rat kidney (original magnification 40 ×). In these cases there is also reactivity to the parietal cells of the rat stomach.

Three types of anti-LKM autoantibodies have been identified [3,5,14,15,18,30,63,72,85]. The LKM type 1 autoantibody (anti-LKM-1) is the characteristic serologic marker for the diagnosis of AIH-2 [5,18,63,72]. These autoantibodies were first described by Rizzetto et al [86], using the IIF method on rodent liver and kidney sections. The characteristic features of anti-LKM-1 autoantibodies are the diffuse staining of cytoplasm of the entire liver lobule and the exclusive staining of the P3 portion of the proximal renal tubules (Fig. 6) [18]. Due to this staining pattern of kidney sections anti-LKM-1 can be easily distinguished from AMA, which stain proximal and distal renal tubules (Fig. 7). Western blots with hepatic and renal microsomes revealed a protein band at 50 kDa [5,14,15,63,72,87].

The major target-autoantigen of anti-LKM-1 autoantibodies in AIH-2 has been identified as the cytochrome P450 2D6 (CYP2D6) [87-89]. It has been shown that anti-LKM-1 autoantibodies inhibit the enzymatic activity of CYP2D6 in vitro, but not in vivo [90]. Epitope mapping experiments of CYP2D6 autoantigen have defined at least four different linear epitopes [91,92]. The most immunodominant epitopes of CYP2D6 were amino acids 257–269 and 321–351, which are recognized in about 70% and 50% of AIH-2 cases, respectively [91,92]. Two infrequent epitopes consisting of amino acids 373–389 and 410–429 are also recognized by anti-LKM-1 in some cases [92]. Recently, Klein et al [93] and Kerkar et al [94] reported another immunodominant epitope of CYP2D6 (amino acids 193–212) recognized in about 70% and 93 % of AIH-2 patients, respectively. However, due to failure of inhibition of CYP2D6 enzymatic activity using epitope specific antibodies and since the absorption of the above linear epitopes was unable to absorb inhibitory anti-CYP2D6 autoantibodies, the existence of additional conformational epitopes on CYP2D6 autoantigen has been postulated [95].

It is noteworthy to state here that depending on the geographic origin, 0–7% of patients with chronic hepatitis C – irrespective of the HCV genotype – develop anti-LKM-1 autoantibodies [6,14,43,63,96,97]. Recently, two studies have shown a higher prevalence of anti-LKM autoantibodies (up to 10%) in a small number of children or adult patients with HCV infection [98,99]. As stated for AIH-2, CYP2D6 is the major target autoantigen recognized by anti-LKM-1 autoantibodies in HCV patients [14,15,81-83,88,92-96]. However, we and others have failed to document CYP2D6 as the major target autoantigen of anti-LKM antibodies in HCV/anti-LKM positive sera [98,99]. In addition, recently Miyakawa et al [100] identified CYP2E1 and CYP3A4 as target autoantigens of anti-LKM autoantibodies in two patients with anti-LKM-positive chronic hepatitis C. Taking together, these findings may further indicate the heterogeneous autoimmune reactions that might take place in anti-LKM positive patients with chronic hepatitis C.

The antigenic sites on CYP2D6 autoantigen recognized by anti-LKM-1 autoantibodies are different in AIH-2 and HCV/anti-LKM-1 positive cases [92-95,101-104]. For example, the major linear epitope of 257–269 amino acids, as well as the newly reported peptide of 193–212 amino acids are recognized in 70–93% of AIH-2 patients but only in 18–50% of HCV/anti-LKM-1 positive patients [83,93,94,101]. Additional support to the presence of conformation-dependent anti-LKM-1 autoantibodies in HCV/anti-LKM-1 positive serum samples has emerged from previous studies [99,102,105]. In the latter studies only about 30% of HCV/anti-LKM-1 positive sera reacted with 50 kDa component using Western blot assays, while additional bands at 59 kDa, 70 kDa and 80 kDa were detected [99,102,105]. However, even taking into account the above additional bands, no more than 45% of all sera tested reactive by Western blot. In contrast, a significant proportion of the previous negative sera tested positive for anti-LKM-1 using a specific competitive ELISA, while denaturation of the antigens prior to perform the ELISA resulted in complete loss of the signal [105].

Recently, the development of a more sensitive and specific assay for the detection of anti-LKM-1 autoantibodies was achieved [14,15]. This novel assay is a quantitative RLA based on immunoprecipitation using ^35^S-methionine-labelled CYP2D6 antigen obtained by in vitro transcription and translation synthesis [81-83,99,104]. Using this assay it was shown that the anti-LKM-1 titers do not differ significantly between AIH-2 and HCV/anti-LKM-1 positive patients [81-83]. The presence of anti-LKM-1 in some patients with HCV infection led to the proposal for a further division of AIH-2 into AIH-2a (younger, predominantly female patients without evidence of HCV infection) and AIH-2b (older, predominantly male patients with HCV infection) [13,106]. Nowadays however, after the marked improvements in the reliability and availability of tests for HCV detection such a subdivision of AIH-2 appears unreasonable and tends to be deleted. Actually, HCV/anti-LKM-1 positive patients represent cases of "true" HCV infection with autoimmune features [6,107].

From the clinical point of view, screening for anti-LKM autoantibodies is recommended before the initiation of interferon-alpha therapy in HCV patients and if found positive a careful monitoring appears reasonable because occasionally interferon-alpha may unmask, or provoke autoimmune hepatic reactions and even "true"AIH [6,43,104,108-110]. Dalekos et al [104] studied antibody titers and performed epitope mapping of LKM-1-positive sera from patients with chronic hepatitis C. Interestingly, a patient with a high LKM-1 titer and autoantibodies directed against an epitope of amino acids 257–269, which are preferentially recognized by patients with AIH-2, showed exacerbation of the disease under interferon-alpha treatment. In contrast to other patients with HCV infection, this patient further recognized a rarely detected epitope on the C-terminal third of the protein. These results suggest that determination and monitoring of CYP2D6 autoantibody titers by both IIF and the RLA in combination with epitope mapping of CYP2D6 in HCV/anti-LKM positive patients before the initiation of interferon-alpha treatment, might be helpful in an attempt to identify those patients at risk of developing undesired autoimmune reactions [104].

**Figure 8:**
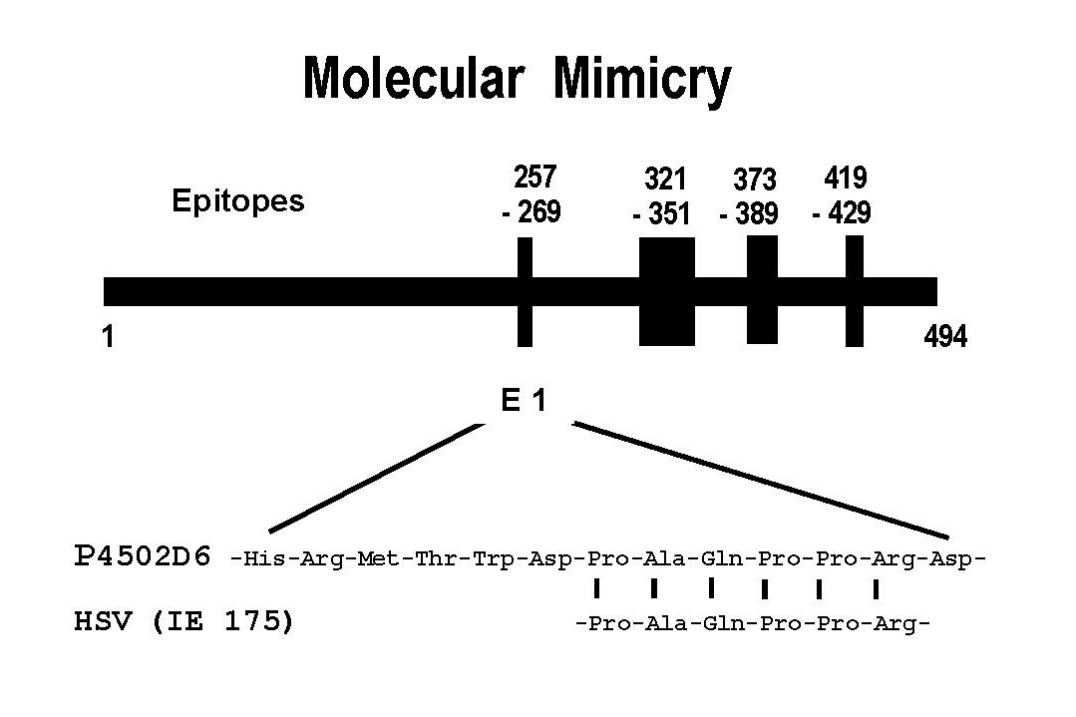
Linear B-cell epitopes on cytochrome P450 2D6 in autoimmune hepatitis type 2. The immunodominant epitope 257–269 aa shares sequence homology with the immediate early protein IE 175, a transcription factor of herpes simplex virus type 1 (now known as infected cell protein 4 or ICP4). Although this is an attractive model for the hypothesis of molecular mimicry, overall evidence for mimicry as a driving force of autoimmune hepatitis is not convincing.

The mechanism(s) of the development and the pathogenic role of anti-LKM-1 autoantibodies in hepatocellular injury are still unclear. It has been suggested that viral infections by herpes simplex virus (HSV) and related viruses may trigger the autoantibody formation through molecular mimicry in at least some individuals with AIH-2 [91]. Manns et al [91] tested 26 LKM-positive sera using Western blot with partial sequences of recombinant CYP2D6. Eleven sera recognized a short minimal epitope of eight amino acids with the sequence DPAQPPRD. Twelve other clones recognized a larger epitope containing this eight-amino acid core sequence. The search of electronic databases revealed an interesting match of the minimal epitope with the primary structure of the immediate early protein IE 175 of HSV-1 now known as infected cell protein 4 (ICP4) of HSV-1 (Fig. 8). Sequence identity was present for the PAQPPR sequence. This hypothesis was further supported by a case study in a pair of identical twins [111]. In this study, one sister suffered from AIH-2 but the other one was healthy. Interestingly, only the sister suffering from AIH-2 was HSV positive, and her serum recognized the viral protein ICP4 in lysates of HSV-infected cells [111]. So far however, overall evidence for mimicry as a driving force of AIH is not convincing.

Besides molecular mimicry, chemical modification of self-proteins and/or immunological cross-reactivity to homologous autoantigens may also provide potential triggers for autoimmune responses. The latter has been suggested by Choudhuri et al [112] who have shown that in AIH-2 patients the linear epitope 321–351 of CYP2D6 cross reacts with amino acids 33–51 of carboxypeptidase-H (the target autoantigen of islet cell autoantibodies in insulin dependent diabetes mellitus), as well as, with amino acids 307–325 of 21-hydroxylase (major target autoantigen in Addison's disease). These findings possibly indicate the presence of a common motif of CYP2D6, carboxypeptidase-H and 21-hydroxylase, which may contribute through a cross reactive immune response to the development of multiple endocrinopathies in the course of AIH-2. Additional support to this hypothesis emerged from two recent studies by Kerkar et al [94] and Bogdanos et al [113]. In the first study the authors were able to show the similarity and cross-reactivity between the immunodominant epitope 193–212 of CYP2D6 and homologues of two unrelated viruses (HCV 2977–2996 and CMV 121–140) [94]. In the second study the researchers investigated whether the immunodominant epitope 252–271 of CYP2D6 in anti-LKM-1 positive AIH-2 and homologues from the NS5B and E1 proteins of the HCV polyprotein and the ICP4 of HSV-1 are targets of humoral immune response in anti-LKM-1 positive and anti-LKM-1 negative HCV infected patients and furthermore whether this response is cross-reactive [113]. The hypothesis of molecular mimicry and cross-reactivity in LKM-1 production has not been addressed experimentally. The authors for the first time gave experimental support to the notion that molecular similarity between CYP2D6, HCV and HSV can result in LKM-1 production via a cross-reactive response in genetically susceptible individuals (interestingly only the HCV positive/LKM-1 positive patients with viral/self cross-reactivity possessed the HLA B51 allotype) [113]. Taking together, the above studies suggest that multiple exposure to viruses mimicking self may represent an important pathway to the development of autoimmunity [94,113].

Two possible mechanisms have been proposed for the involvement of anti-LKM-1 autoantibodies in the pathogenesis of liver injury. The first appears to be a direct binding of these autoantibodies to hepatocytes, leading to lysis of liver cells, while the second is associated with an anti-LKM-1 induction of activating liver-infiltrating T lymphocytes, which indicates the combination of B and T cell activity in the autoimmune process involved [114-117]. A prerequisite for both anti-LKM-1 production and the activation of pathogenetic mechanisms involved in liver injury, is the expression of CYP2D6 on the surface of the patients' hepatocytes. Under this context, Ma et al [118] showed that key residues of a major CYP2D6 epitope (316–327) are exposed on the surface of the molecule and may represent key targets for anti-CYP2D6 production. In addition, recent data provides convincing evidence that anti-LKM-1 autoantibodies recognize CYP2D6 exposed on the plasma membrane of hepatocytes from either AIH-2 or HCV/anti-LKM-1 positive patients [114,115] suggesting a pathogenetic role for these autoantibodies in hepatic tissue damage either in AIH-2 or in some cases of HCV/anti-LKM-1 positive patients [104,109,110,115].

So far, anti-LKM type 2 autoantibodies (anti-LKM-2) have been detected only in some cases of drug-induced hepatitis caused by tienilic acid [14,63]. The target autoantigen of anti-LKM-2 has been documented as the CYP2C9 [85]. A proposed mechanism for the induction of anti-LKM-2 could be the binding of an active metabolite of the drug to the CYP2C9 protein, which then becomes antigenic [14,63,72,85].

Anti-LKM type 3 autoantibodies (anti-LKM-3) alone or in combination with anti-LKM-1 are also detected in about 5–10% of patients with AIH-2 [16,119]. In contrast to anti-LKM-1 and anti-LKM-2 autoantibodies, which on immunofluorescence stain liver and kidney tissues only, with anti-LKM-3 additional fluorescence signals may be present with tissue from the pancreas, adrenal gland, thyroid, and stomach. Family 1 of UDP-glycuronosyltransferases (UGT1) is the main target autoantigen of anti-LKM-3 autoantibodies (molecular weight of 55 kDa) [119,120]. These autoantibodies were first described in about 13% of patients with chronic hepatitis D, but not in patients with chronic hepatitis B or C [121]. However, three recent reports have shown the presence of anti-LKM-3 autoantibodies in some patients with HCV infection [99,122,123]. These findings may further support the heterogeneous phenomenon of the HCV-induced autoimmunity.

### 4.2. Autoantibodies against liver cytosolic protein type 1 (anti-LC1)

In 1988 a second autoantibody marker of AIH-2 was recognized [19]. This autoantibody was found to react to a liver cytosolic protein. The autoantibody is organ specific but not species specific and was therefore called anti-LC1 [19]. The anti-LC1 autoantibodies are characterized by a cytoplasmic staining of the periportal hepatocytes when the IIF assay is used for their detection. The hepatocellular layer around the central veins is not stained [19,124]. These findings indicate that the target autoantigen of anti-LC1 autoantibodies is not uniformly distributed in rodent liver tissues. They are detected in about 30% of patients with AIH-2 [19,124] and in approximately 50% of all anti-LKM-1 positive cases [125]. It is noteworthy that the anti-LC1 autoantibodies proved to be the only serological marker in 10% of patients with AIH [19].

The detection of anti-LC1 autoantibodies by IIF is obscured due to the anti-LKM-1 pattern that frequently found in most of the anti-LC1 positive sera. For these reasons other techniques such as, the ouchterlony double diffusion, immunoblot or counter-immunoelectrophoresis are required for their detection [19,124-126]. By immunoblotting, anti-LC1 positive serum samples recognize a liver specific cytosolic protein of 58–62 kDa [124-126]. Recently the molecular target of anti-LC1 was identified as the formiminotransferase cyclodeaminase (FTCD) [127], which is a polymeric bifunctional enzyme involved in folate metabolism. However, another group demonstrated the arginninosuccinate lyase (ASL) as the target autoantigen of a weak precipitin line detected by the ouchterlony double diffusion assay in patients with autoimmune or viral hepatitis [128].

Anti-LC1 autoantibodies have been proposed as a more specific marker of AIH-2 than anti-LKM-1 autoantibodies, since in the original reports their presence was never associated with HCV infection [19,124]. However, a recent study by Lenzi et al [125] confirmed the above aspect only in the pediatric subset of their patients, while a substantial proportion of the adults with anti-LC1 autoantibodies had also markers of HCV infection. The significance of the association between anti-LC1 autoantibodies and HCV infection remains uncertain and has to be established [106,129]. In contrast to what has been found for anti-LKM-1, the titers of anti-LC1 autoantibodies appear to parallel with disease activity [130]. The latter may indicate a possible involvement of anti-LC1 in the pathogenesis of AIH-2. However, the clinical significance of anti-LC1 is not yet completely defined.

## 5. Detectable Autoantibodies in AIH in APECED

Chronic hepatitis as a disease component of APECED may develop in 10–18% of patients [14,15,23-25,28,29,63]. APECED appears to be caused by mutations in a recently identified gene, the autoimmune regulator gene (AIRE), and represents the only known autoimmune disease with a monogenetic mutation today [26,27,131]. It is interesting that patients with AIH in the absence of APECED do not display mutations of the AIRE gene and are therefore genetically distinct from patients with AIH as a component of APECED [132].

Similar to AIH-2, hepatitis in APECED is associated with autoantibodies directed against cytochrome P450 proteins. In a large study with APECED patients, a typical LKM staining pattern and a predominant staining of the perivenous hepatocytes in the absence of staining of the kidney were observed [23]. The latter pattern is due to autoantibodies called liver microsomal autoantibodies (anti-LM). In this study each of anti-LKM and anti-LM antibodies were found in 8% of the patients [23]. These findings indicate that two or more different microsomal antigens are hepatic target-autoantigens in APECED.

Indeed, screening of APECED sera with recombinant antigens using Western blots has shown reactivity against four different hepatic cytochromes P450: CYP1A1, CYP1A2, CYP2A6 and CYP2B6 [23-25,133]. CYP1A1, CYP2A6 and CYP2B6 are expressed both in liver and in kidney resulting to an LKM staining pattern, while CYP1A2 is not expressed in the kidney leading to the LM staining. Among the four autoantibodies anti-CYP2A6 were detected with the highest prevalence in a Finnish APECED patients group (15.6%), while anti-CYP1A2 were found in 6.3% [23]. These results were confirmed by quantitative immunoprecipitation assays with recombinant ^35^S-labeled CYP1A2 and CYP2A6.

Contrary to a previous work in Sardinian patients with APECED [25], the detection of anti-CYP2A6 autoantibodies in a larger group of Finnish patients was not associated with the presence or absence of hepatitis, while anti-CYP1A2 autoantibodies were detected only in APECED patients with hepatitis [23]. These findings indicate that anti-CYP1A2 is a specific marker for AIH as a component of APECED, albeit with a low sensitivity [23,24]. Anti-CYP2A6 autoantibodies may be used as an indicator for APECED, if they are present in a patient with AIH. In parallel with the above conclusions is the anti-LKM/LM detection by IIF in about 50% of patients with AIH as part of the APECED and in only 11% of APECED patients without hepatitis [23]. The same study showed that the prevalence of ANA detection in APECED patients was high (22%) but irrespective of the presence or absence of hepatitis. Therefore ANA are not useful laboratory markers for AIH in APECED [23]. To the contrary, none of the patients' sera tested positive for anti-SLA, anti-CYP2D6 or anti-FTCD autoantibodies, which are specific markers of AIH-1 and AIH-2 [23]. On the other hand, CYP1A2 and CYP2A6 could not be identified as hepatic autoantigens in the disease control groups consisting of patients with idiopathic AIH or patients with autoimmune rheumatic diseases [23]. These findings indicate that idiopathic AIH and AIH in APECED are characterized by different molecular targets of autoimmunity, which do not overlap. Therefore, AIH and hepatitis as part of the APECED may be distinguished on the basis of differences in autoantibody profile (Tables 4 and 5).

**Table 4:** Detectable autoantibodies in AIH-1, AIH-2 and AIH as part of APECED

*AIH-1 or AIH-2*	*AIH in APECED*
ANA, SMA, ANCA, anti-ASGP-R anti-SLA/LP (molecular target: UGA suppressor tRNA-associated protein), anti-LKM-1 (molecular target: CYP2D6), anti-LKM-3 (molecular target: UGT1), anti-LC1 (molecular target: FTCD)	ANA, anti-LC (molecular target: unknown), anti-LKM (molecular targets: CYP2A6, CYP1A1 and CYP2B6), anti-LM (specific autoantibody; molecular target: CYP1A2)

Abbreviations are the same as shown in the text.

**Table 5:** Differential diagnosis of chronic liver diseases according to the presence or absence of autoantibodies against molecularly defined autoantigens of cytochrome P450 complex using the radioligand assay.

*Anti-CYP2D6*	*Anti-CYP2A6*	*Anti-CYP1A2*	*Chronic liver disease*
+	-	-	AIH-2 (94–100%), HCV (0–10%)
-	+	-	HCV, APECED with or without hepatitis
-	-	+	AIH in APECED, drug induced hepatitis
+	+	-	HCV (0–7%)
-	+	+	AIH in APECED

Abbreviations are the same as shown in the text.

Dalekos et al [134] using the sensitive quantitative RLA reported for the first time the presence of anti-CYP2A6 autoantibodies in about 2% of HCV-positive sera in general and in 7.5% of LKM-1-positive/HCV-positive sera. The latter further supports the low specificity of this autoantibody as a marker for AIH in APECED. Interestingly, anti-CYP2A6 autoantibodies were not detected in patients with AIH-2 who exhibit high titers of anti-LKM-1 autoantibodies [134]. The clinical relevance of this finding in HCV infection remains to be determined.

Anti-LM autoantibodies are first described in dihydralazine-induced hepatitis [135]. The major target autoantigen of anti-LM in both conditions (hepatitis as part of the APECED and drug-induced hepatitis) has been documented as the CYP1A2 [23-25,133,135]. In cases of dihydralazine-induced hepatitis the production of anti-LM autoantibodies has been attributed to adduct formation of CYP1A2 with an activated metabolite of the drug [136]. By contrast, in APECED patients no relationship between CYP1A2 and drug usage is known. In addition, it is not known whether in APECED patients a close monitoring of anti-LM may lead to early, or even prophylactic, treatment of hepatitis as a new disease component. Evidence that autoantibodies may be found before the clinical and/or laboratory manifestation of a new disease component in APECED comes from adrenal and ovarian insufficiencies, where the respective autoantibodies are detected 2–3 years before the clinical presentation of the autoimmune components [137].

Another hepatic autoantigen in APECED, the aromatic-L-amino acid decarboxylase (AADC) has also been identified recently [133,138]. This enzyme is expressed in the liver cytosol and was originally described as a β-cell autoantigen [133]. The prevalence of anti-AADC autoantibodies is significantly increased in APECED patients with vitiligo (88%) and hepatitis (92%) [5,14,29]. So far, anti-AADC autoantibodies have only been reported in APECED and their role in AIH and vitiligo as disease components of APECED deserve further investigation.

## 6. Concluding Remarks

In clinical practice the recognition of AIH is of great importance since most of the patients respond favorably to antiinflammatory and immunosuppressive treatment. In addition, recent novel findings dealing with the bone marrow hemopoietic progenitor cells and bone marrow stromal cells of patients with AIH suggests alternative therapeutic options even in refractory cases [139]. Diagnostic criteria for this disease have been codified recently [6]. These include descriptive criteria and scoring system based on clinical, serologic and histologic features of AIH (Table 1), which contribute substantially to the differential diagnosis of the disease from other forms of chronic hepatitis associated with autoimmune phenomena (Table 6). The discrimination between AIH and HCV infection is of particular importance, since the immunosuppression used in the former can deteriorate liver disease in HCV patients, while interferon-alpha treatment used in HCV infection may lead to exacerbation of AIH [104,108-110].

**Table 6:** Differential Diagnosis of Autoimmune Hepatitis.

Other autoimmune liver diseases
- Overlap syndromes
- Primary biliary cirrhosis
- Primary sclerosing cholangitis
Chronic viral hepatitis
- Chronic hepatitis B with or without hepatitis delta
- Chronic hepatitis C
- Chronic hepatitis non A to G
Cholangiopathy due to human immunodeficiency virus infection
Alcoholic liver disease
Drug-induced hepatitis
Non-alcoholic steatohepatitis
Granulomatous hepatitis
Hemochromatosis
α_1_-antithrypsin deficiency
Wilson's disease
Systemic lupus erythematosus

The detection of non-organ specific autoantibodies remains the hallmark of AIH. A step by step diagnostic application of autoantibody tests is mandatory for the evaluation of acute or chronic hepatitis of unknown cause. ANA, SMA and anti-LKM-1 autoantibodies should be first tested in patients with acute or chronic elevation of aminotransferases when virologic tests are negative and there is no current or past history for drug or alcohol abuse. Determination of ANCA, which occur in up to 90% of patients with AIH-1, may be useful in the identification of individuals who are seronegative for the above conventional autoantibody markers but should be kept in mind that this autoantibody lacks specificity. Many target autoantigens of the non-organ specific autoantibodies have been identified, but the latter has not led to the characterization of specific subpopulations of patients or changes in the treatment strategies. In addition, most of the non-organ specific autoantibodies do not seem to be involved in the pathogenesis of liver injury in AIH. Anti-LKM-1 autoantibodies could be an exception to the above aspect since recent data have demonstrated the expression of CYP2D6 on the surface of hepatocytes, while AIH-2 has not been observed in individuals who are deficient for CYP2D6. These findings provide arguments for an antigen-driven autoimmune process. It is possible that mutations in the autoantigen itself can lead to alterations in the three dimensional structure of the antigen, which induces autoimmunity.

Antibodies directed against liver-related antigens have had similar limitations. Anti-ASGP-R and anti-LC1 autoantibodies appear to correlate with disease severity and response to treatment suggesting a pathogenetic role to the hepatocellular damage. In general however, autoantibodies should not be used as a tool for monitoring of treatment or to predict AIH activity and outcome. Anti-SLA/LP autoantibodies have been considered as valuable and specific markers for the diagnosis of AIH-1. However, a recent study has shown that anti-SLA/LP autoantibodies can also be detected in AIH-2 and in children with PSC. Irrespective of the disease specificity of this marker, it is obvious that testing for anti-SLA/LP will help to reduce the group of cryptogenic liver disease, by recognizing previously misdiagnosed patients with AIH who were seronegative for ANA, SMA or anti-LKM-1.

In APECED, autoantibodies are directed against specific cytochrome P450 enzymes (e.g. CYP1A2, CYP2A6, CYP21, CYP17, and CYP11A1), that are expressed in organs affected by the disease process. These observations argue against the idea that antibodies against cytochrome P450 complex are simply epiphenomena secondary to tissue damage and that they have no relation to the etiology and pathogenesis of APECED.

It is not known what triggers autoimmunity in AIH. The hypothesis that different causes may lead to loss of tolerance against the same molecular target autoantigen seems attractive. For instance, CYP1A2 is the molecular target in dihydralazine-induced hepatitis and AIH as a component of APECED, CYP2D6 in AIH-2 and in some patients with HCV infection, CYP2A6 in APECED and in a proportion of patients with HCV infection and UGT1 in some cases of AIH-2 and chronic hepatitis D or C.

Research protocols in order to define AIH pathogenesis, disease susceptibility, determinants of disease severity, and to understand the epidemiology of AIH are future challenges in the investigational and clinical arena of this disease [139-141].

## Competing interest

The author(s) declare that they have no competing interests.
